# Clinicopathological heterogeneity between primary and metastatic sites of gastroenteropancreatic neuroendocrine neoplasm

**DOI:** 10.1186/s13000-020-01030-x

**Published:** 2020-09-11

**Authors:** Huiying Shi, Chen Jiang, Qin Zhang, Cuihua Qi, Hailing Yao, Rong Lin

**Affiliations:** 1 Department of Gastroenterology, Union Hospital, Tongji Medical College, Huazhong University of Science and Technology, Wuhan, 430022 China; 2 Department of Pathology, Union Hospital, Tongji Medical College, Huazhong University of Science and Technology, Wuhan, 430022 China

**Keywords:** Gastroenteropancreatic neuroendocrine neoplasms (GEP-NENs), Chromogranin (CgA), Synaptophysin (Syn), Ki-67 index, Metastasis, Heterogeneity

## Abstract

**Background:**

Chromogranin A (CgA), synaptophysin (Syn) and the Ki-67 index play significant roles in diagnosis or the evaluation of the proliferative activity of gastroenteropancreatic neuroendocrine neoplasms (GEP-NENs). However, little is known about whether these biological markers change during tumor metastasis and whether such changes have effect on prognosis.

**Methods:**

We analyzed 35 specimens of both primary and metastatic tumor from 779 patients who had been diagnosed as GEP-NENs at Wuhan Union Hospital from August 2011 to October 2019. The heterogeneity of CgA, Syn and Ki-67 index was evaluated by immunohistochemical analysis.

**Results:**

Among these 779 patients, the three most common sites of NENs in the digestive tract were the pancreas, rectum and stomach. Metastases were found in 311 (39.9%) patients. Among the 35 patients with both primary and metastatic pathological specimens, differences in the Ki-67 level were detected in 54.3% of the patients, while 37.1% showed a difference in CgA and only 11.4% showed a difference in Syn. Importantly, due to the difference in the Ki-67 index between primary and metastatic lesions, the WHO grade was changed in 8.6% of the patients. In addition, a Kaplan–Meier survival analysis showed that patients with Ki-67 index variation had a shorter overall survival (*p* = 0.0346), while neither Syn variation nor CgA variation was related to patient survival (*p* = 0.7194, *p* = 0.4829).

**Conclusions:**

Our data indicate that primary and metastatic sites of GEP-NENs may exhibit pathological heterogeneity. Ki-67 index variation is closely related to the poor prognosis of patients with tumor metastasis, but neither Syn variation nor CgA variation is related to patient prognosis. Therefore, clinicopathologic evaluation of the primary tumor and metastatic sites could be helpful for predicting the prognosis.

## Introduction

Neuroendocrine neoplasms (NENs) are a group of rare and highly heterogeneous neoplasms originating from peptidergic neurons and neuroendocrine cells, which can exist in all parts of the body [1]. Recently, the incidence of NENs has been on the rise. The actual incidence rate increased 6.4-fold from 1973 (1.09 per 100,000) to 2012 (6.98 per 100,000) according to data from the National Cancer Institute’s Surveillance, Epidemiology, and End Results (SEER) Program [2]. Gastroenteropancreatic neuroendocrine neoplasms (GEP-NENs) are reportedly the most common of these, and account for about 66% of all NENs [3]. In addition, 21 to 73.4% of GEP-NENs were found to have metastases at the time of diagnosis [4–6].

Chromogranin A (CgA) and synaptophysin (Syn) widely exist in neuroendocrine cells, and are currently necessary markers for the diagnosis of NENs [7, 8]. CgA was initially found in chromaffin particles of adrenal medulla containing catecholamines [9]. It is a kind of acidic hydrophilic secreted protein found in the secretory vesicles of neuroendocrine cells [10]. Syn is an integral membrane glycoprotein present in presynaptic neuron vesicles and the vesicles of normal and neuroendocrine tumor cells [7, 11]. In addition, the Ki-67 index plays a crucial role in the World Health Organization (WHO) grading system for GEP-NENs: (grade 1) G1 tumors have a Ki-67 index < 3%; (grade 2) G2 tumors have an index of 3–20%; and (grade 3) G3 tumors have an index > 20% [12]. Syn, CgA and the Ki-67 index play important roles in the diagnosis of NENs. However, little is known about whether these compounds change during tumor metastasis and whether such changes play a role in the overall process of metastasis.

Therefore, the purposes of this study were to investigate the clinicopathological heterogeneity of CgA, Syn, and the Ki-67 index between the primary and metastatic sites, and to evaluate the influence of these clinicopathological features on the prognosis in patients with metastatic GEP-NENs.

## Materials and methods

### Patients and data collection

This study retrospectively collected and analyzed data from 779 patients diagnosed with GEP-NENs at Wuhan Union Hospital from 2011 to 2019; specimens of both primary and metastatic sites were available for 35 patients. The diagnosis of NENs mainly depended on the histological morphology as well as the immunohistochemical staining results of various neuroendocrine markers like CgA and Syn. NENs could also be diagnosed by CD56 or neuron-specific enolase when the immunostaining result of CgA or Syn was absent [13]. All specimens were obtained by surgical excision, fine needle aspiration, and/or core biopsy. Patients for whom complete clinical data were not available for extraction were excluded. The following demographic and clinical characteristics of the patients were collected: age, sex, primary and metastatic tumor sites, immunohistochemical results (CgA, Syn and Ki-67 index), and survival time. The grading system used in this study was based on the WHO 5th edition classification (2019) of digestive system tumors, in which NENs are classified as well-differentiated neuroendocrine tumors (NETs), poorly differentiated neuroendocrine carcinomas (NECs), and mixed neuroendocrine–non-neuroendocrine neoplasm (MiNEN). NETs were further divided into three grades based on the Ki-67-positive index: G1: < 3%, G2: 3–20%, G3: > 20% [12]. According to the AJCC cancer staging manual, if the metastasis occurred simultaneously with the primary tumor or within 4 months after the initial resection of the primary tumor, the metastasis was considered to be synchronous; if the metastasis occurred more than 4 months after the initial resection of the primary tumor, it was considered as metachronous [14].

The study was approved by the Ethics Committee of Tongji Medical College, Huazhong University of Science and Technology (IORG number: IORG0003571) and was conducted in accordance with the Declaration of Helsinki.

### Immunohistochemistry

We performed immunohistochemical staining for CgA, Syn and Ki-67. The primary and metastatic tumor tissues were fixed in 4% paraformaldehyde and embedded in paraffin blocks. Each block was cut into 4 μm-thick section, and deparaffinized with xylene and rehydrated with ethanol. Then the sections were incubated with hydrogen peroxide at the room temperature, then antigen retrieval and serum blocking were performed. After these steps, they were incubated with the CgA (M0869, Dako, Glostrup, Denmark), Syn (M7315, Dako) and Ki-67 antibodies (MIB-1, DAKO, Agilent Technologies, Santa Clara, CA, USA) at 37°Cfor 1 h. Then they were incubated with the secondary antibody containing the biotin. Last, slides were visualized by using the DAB (AR1022, BOSTER Biological Technology), stained with hematoxylin, dehydrated with ethanol, transparentized with xylene, and counted.

The staining results for CgA and Syn were graded according to the extent of positive cells as follows: grade I (negative, less than 5% positive tumor cells), grade II (focally positive, 5 to 50% positive tumor cells), grade III (positive, more than 50% positive tumor cells) [15]. For Ki-67 staining, the areas with abundant and more positive tumor cells were selected. The Ki-67 labeling index was determined by calculating the percentage of positive nuclei of 2000 tumor cells in the densest area of each slide [16]. Each slide was reexamined by the pathologist (Q.Z.).

### Statistical analysis

SPSS software v24.0 (IBM, Armonk, NY, USA) or GraphPad Prism v6.0c (GraphPad Software, San Diego, CA, USA) was used for statistical calculation and data processing. The clinicopathological characteristics of patients were expressed as median and range, absolute value or fractions. Overall survival (OS) was defined as the date of initial diagnosis to the last follow-up or date of death. Survival curves were drawn according to the Kaplan–Meier analysis, and differences between groups were assessed using the log-rank test. *P* < 0.05 was regarded as statistically significant.

## Results

### Patient clinical and pathological characteristics

A total of 779 patients with GEP-NENs were included in the analysis; their clinicopathological characteristics were shown in Table 1. There were 460 (59.1%) males and 319 (40.9%) females (male-female ratio of 1.44). The median age at diagnosis was 55 y (range: 13–87 y). Among these 779 patients, the three most common sites of NENs in the digestive tract were the pancreas, rectum and stomach. Pathological diagnosis showed that, among the 779 patients, NET-G1 accounted for 40.2%, NET-G2 18.5%, NET-G3 0.5%, NEC 34.7% and MiNEN 5.1%. Metastasis occurred in 39.9% of the patients (311/779). The positive rates of immunohistochemical staining for CgA and Syn were 57.5 and 85.4%, respectively, and the double-positive rate was 56.6% (441/779). For 35 patients with metastatic specimens, 25 (71.4%) had metastatic tumors simultaneously, and 10 (28.6%) had metachronous metastases.

**Table 1:** Clinical characteristics in 779 patients with GEP-NENs

Variables	Total ***n*** = 779 (%)
**Age at diagnosis (years)**	55 (13–87)
**Sex**	
Male	460 (59.1)
Female	319 (40.9)
**Primary tumor site**	
Pancreas	216 (27.7)
Rectum	208 (26.7)
Stomach	144 (18.5)
Esophagus	44 (5.6)
Duodenum	31 (4.0)
Colon	29 (3.7)
Gallbladder	18 (2.3)
Appendix	11 (1.4)
Small intestine	4 (0.5)
Other sites	74 (9.5)
**Tumor grade and type**	
NET G1	313 (40.2)
NET G2	144 (18.5)
NET G3	4 (0.5)
NEC	270 (34.7)
MINEN	40 (5.1)
Unknown	8 (1.0)
^a^**CgA**	
Positive	448 (57.5)
Negative	221 (28.4)
Unknown	110 (14.1)
^a^**Syn**	
Positive	665 (85.4)
Negative	15 (1.9)
Unknown	99 (12.7)
**Metastasis**	311 (39.9)

*Abbreviations*: *GEP-NENs* gastroenteropancreatic neuroendocrine neoplasms, *NET* neuroendocrine tumors, *NEC* neuroendocrine carcinoma, *MiNEN* mixed neuroendocrine–non-neuroendocrine neoplasm, *CgA* chromogranin; Syn, synaptophysin. ^a^ The expression of CgA or Syn in primary site

### Heterogeneity of CgA, Syn and the Ki-67 index between primary and metastatic sites

**Fig. 1:**
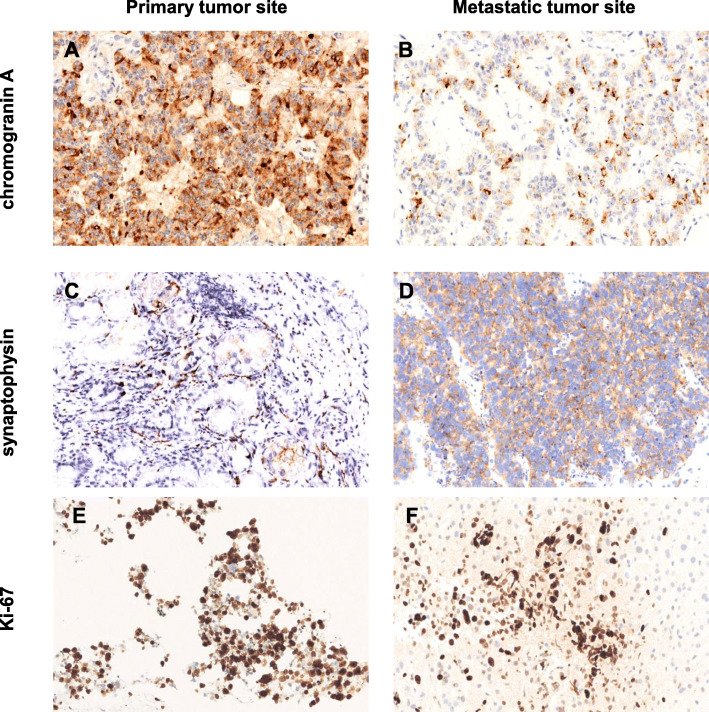
Examples of CgA staining images in the primary pancreatic NEN (**a**) and mesenteric metastasis (**b**) from the patient # 5, magnification 200×. Examples of Syn staining images in the primary gastric NEN (**c**) and hepatic metastasis (**d**) from the patient # 26, magnification 200×. Examples of Ki-67 labeling in the primary pancreatic NEN (**e**) and hepatic metastasis (**f**) from the patient # 6, magnification 200 ×

For 35 patients with both primary and metastatic specimens, the clinicopathological differences between the primary and metastatic sites were analyzed. There was no significant difference in the overall positive rates of CgA and Syn, or in the grading or classification of tumors between the primary and metastatic sites (Supplementary Table 1). However, the variation of CgA, Syn and the Ki-67 index existed between the primary and metastatic sites. Examples of CgA, Syn and the Ki-67 index variation between the primary tumor sites and metastatic sites were shown in Fig. 1.

### CgA

**Fig. 2:**
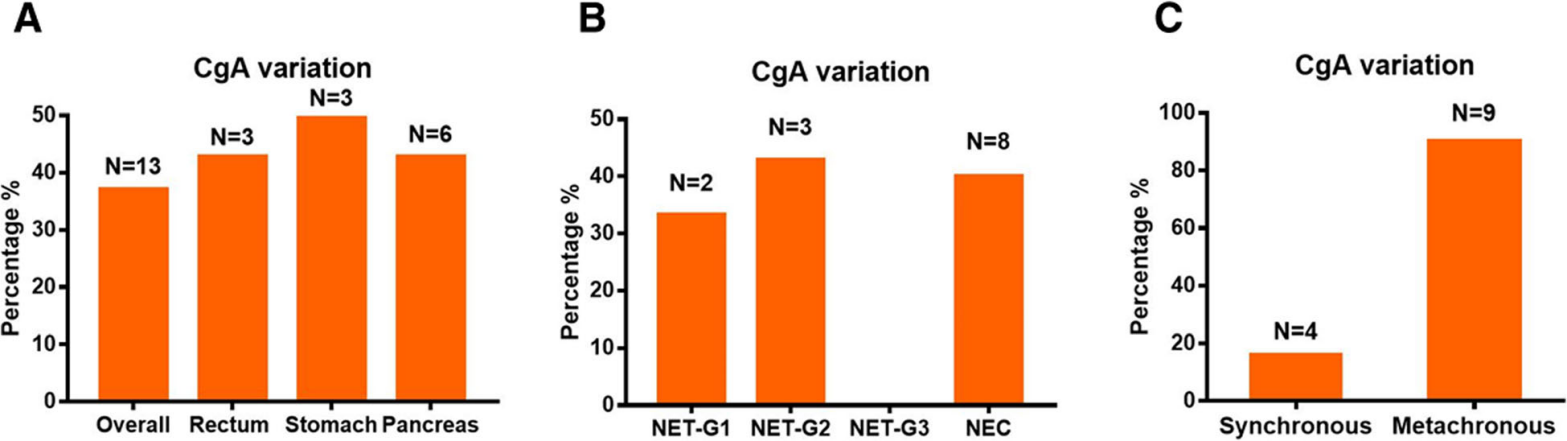
Percentages of patients with CgA-variation at different primary tumor sites (**a**). Percentages of patients with CgA-variation under different classifications of NENs (**b**). Percentages of patients with CgA-variation under different types of metastatic tumors (**c**)

Of the 35 patients, while 18 (51.4%) had no difference in CgA between the primary and metastatic sites, 13 (37.1%) showed a variation in CgA (Table 2). The CgA variation rate was 42.9% (3/7) for rectal NENs, 42.9% (6/14) for pancreatic NENs, and 50% (3/6) for gastric NENs (Fig. 2a). The CgA variation rate was 33.3% (2/6) for NET-G1, 42.9% (3/7) for NET-G2, 0% for NET-G3, and 40% (8/20) for NEC (Fig. 2b). The CgA variation rate was 16% (4/25) for synchronous metastases and 90% (9/10) for metachronous metastases (Fig. 2c).

**Table 2:** CgA, Syn and Ki-67 index variation between primary and metastatic sites

Patient #	Primary tumor site Site1#	Metastatic tumor site Site2#	^a^CgA variability		^a^Syn variability		Ki-67 index(%)		Survival time	WHO class changes
			Site1# Site2#		Site1# Site2#		Site1# Site2#			
1	pancreas	liver	III	III	III	III	1	1	57	–
2	pancreas	liver	III	III	III	III	10	10	75	–
3	pancreas	abdominal wall	I	II	II	I	40	80	83	NEC → NEC
4	pancreas	liver	III	III	III	III	2	/	23	–
5	pancreas	mesentery	III	II	III	III	10	5	12	NET G2 → NET G2
6	pancreas	liver	II	III	III	III	80	60	3	NEC → NEC
7	pancreas	liver	I	I	II	II	30	25	4	NET G3 → NET G3
8	pancreas	liver	III	II	III	III	2	2	1	–
9	pancreas	liver	III	III	III	III	10	10	7	–
10	pancreas	liver	III	III	III	III	40	40	0	–
11	pancreas	liver	III	/	III	III	5	/	3	–
12	pancreas	lymph node	/	/	III	III	30	30	19	–
13	pancreas	liver	I	II	III	III	15	15	27	–
14	pancreas	pelvic cavity	I	II	III	III	10	5	70	NET G2 → NET G2
15	rectum	liver	/	/	III	III	10	10	97	–
16	rectum	liver	III	III	III	III	2	5	27	NET G1 → NET G2
17	rectum	liver	II	I	III	III	2	25	3	NET G1 → NET G3
18	rectum	lymph node	III	I	III	III	60	80	48	NEC → NEC
19	rectum	lymph node	III	II	III	III	60	80	12	NEC → NEC
20	rectum	Liver	I	I	I	III	95	90	20	NEC → NEC
21	rectum	lymph node	I	I	I	III	95	90	20	NEC → NEC
22	stomach	liver	I	I	III	III	70	70	8	–
23	stomach	iliac fossa	III	II	III	III	35	30	16	NEC → NEC
24	stomach	lymph node	I	I	III	III	/	80	15	–
25	stomach	lung	I	II	III	III	70	70	15	–
26	stomach	liver	I	I	II	III	80	50	1	NEC → NEC
27	stomach	liver	III	II	III	III	80	50	36	NEC → NEC
28	duodenum	liver	III	III	III	III	5	2	5	NET G2 → NET G1
29	duodenum	liver	II	II	III	III	5	5	11	–
30	duodenum	adrenal gland	II	II	III	III	20	35	29	NEC → NEC
31	colon	mesentery	II	II	III	III	60	/	55	–
32	unknown	liver	/	/	III	III	80	60	5	NEC → NEC
33	unknown	liver	II	III	III	III	80	90	1	NEC → NEC
34	unknown	peritoneal	II	II	II	II	30	30	21	–
35	unknown	rectum	III	III	III	III	1	2	61	NET G1 → NET G1

*Abbreviations*: *CgA* chromogranin, *Syn* synaptophysin, *NEC* neuroendocrine carcinoma; ^a^CgA and Syn graded according to the percent of positive cells as follows: I: < 5%, II: 5–50%, and III: > 50%

### Syn

**Fig. 3:**
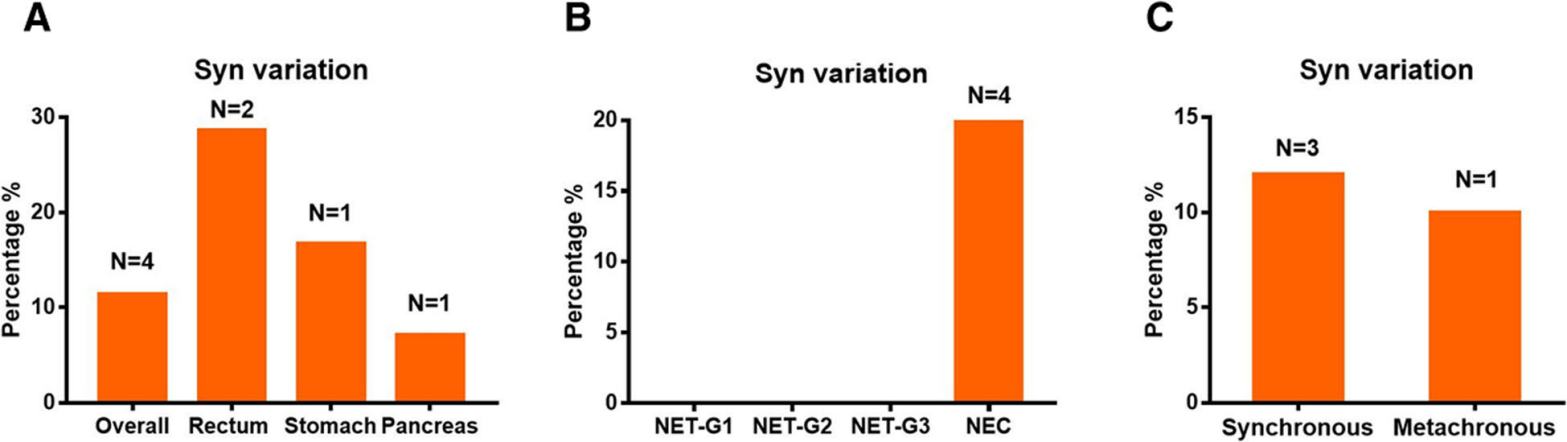
Percentages of patients with Syn-variation at different primary tumor sites (**a**). Percentages of patients with Syn-variation under different classifications of NENs (**b**). Percentages of patients with Syn-variation under different types of metastatic tumors (**c**)

With regard to Syn variation between the primary and metastatic sites, while 31/35 (88.6%) patients had no change, 4/35 (11.4%) patients had variation (Table 2). The Syn variation rate was 28.6% (2/7) for rectal NENs, 7.1% (1/14) for pancreatic NENs, and 16.7% (1/6) for gastric NENs (Fig. 3a). With regard to the WHO classification, Syn variation was observed in 20% (4/20) of NEC, but not in NET-G1, NET-G2, or NET-G3 (Fig. 3b). The Syn variation rate was 12% (3/25) for synchronous metastases and 10% (1/10) for metachronous metastases (Fig. 3c).

### Ki-67 index

**Fig. 4:**
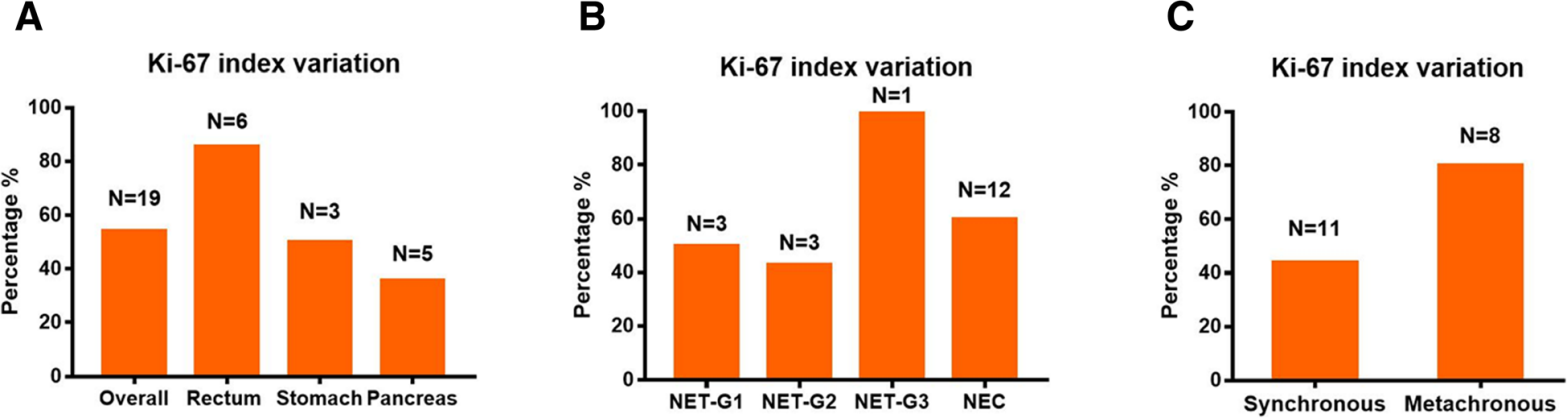
Percentages of patients with Ki-67 index-variation at different primary tumor sites (**a**). Percentages of patients with Ki-67 index-variation under different classifications of NENs (**b**). Percentages of patients with Ki-67 index-variation under different types of metastatic tumors (**c**)

Our results showed that the mean number and standard deviation of Ki-67-positive cells in primary tumor was 37.89 ± 33.04 (%) and the metastatic lesion was 39 ± 27.48 (%). Ki-67 index variation was observed in 19 patients (54.3%); 8 (42.1%) showed up-regulation (from primary to metastasis) and 11 (57.9%) showed down-regulation. In 16 of these 35 patients (45.7%), despite this difference in Ki-67 index between primary and metastatic sites, there was no difference in classification. Notably, for 3/35 patients (8.6%), the classification changed: 1 patient increased from NET-G1 to NET-G2, 1 increased from NET-G1 to NET-G3, and 1 decreased from NET-G2 to NET-G1 (Table 2). The Ki-67 index variation was seen in 85.7% (6/7) of rectal NENs, 50% (3/6) of gastric NENs, and 35.7% (5/14) of pancreatic NENs (Fig. 4a). Moreover, Ki-67 index variation was as high as 60% (12/20) in NEC, 50% (3/6) in NET-G1, 42.9% (3/7) in NET-G2, and 100% in NET-G3 (Fig. 4b). The Ki-67 index variation was observed in 44% (11/25) of synchronous metastases and 80% (8/10) of metachronous metastases (Fig. 4c).

### Survival analysis

**Fig. 5:**
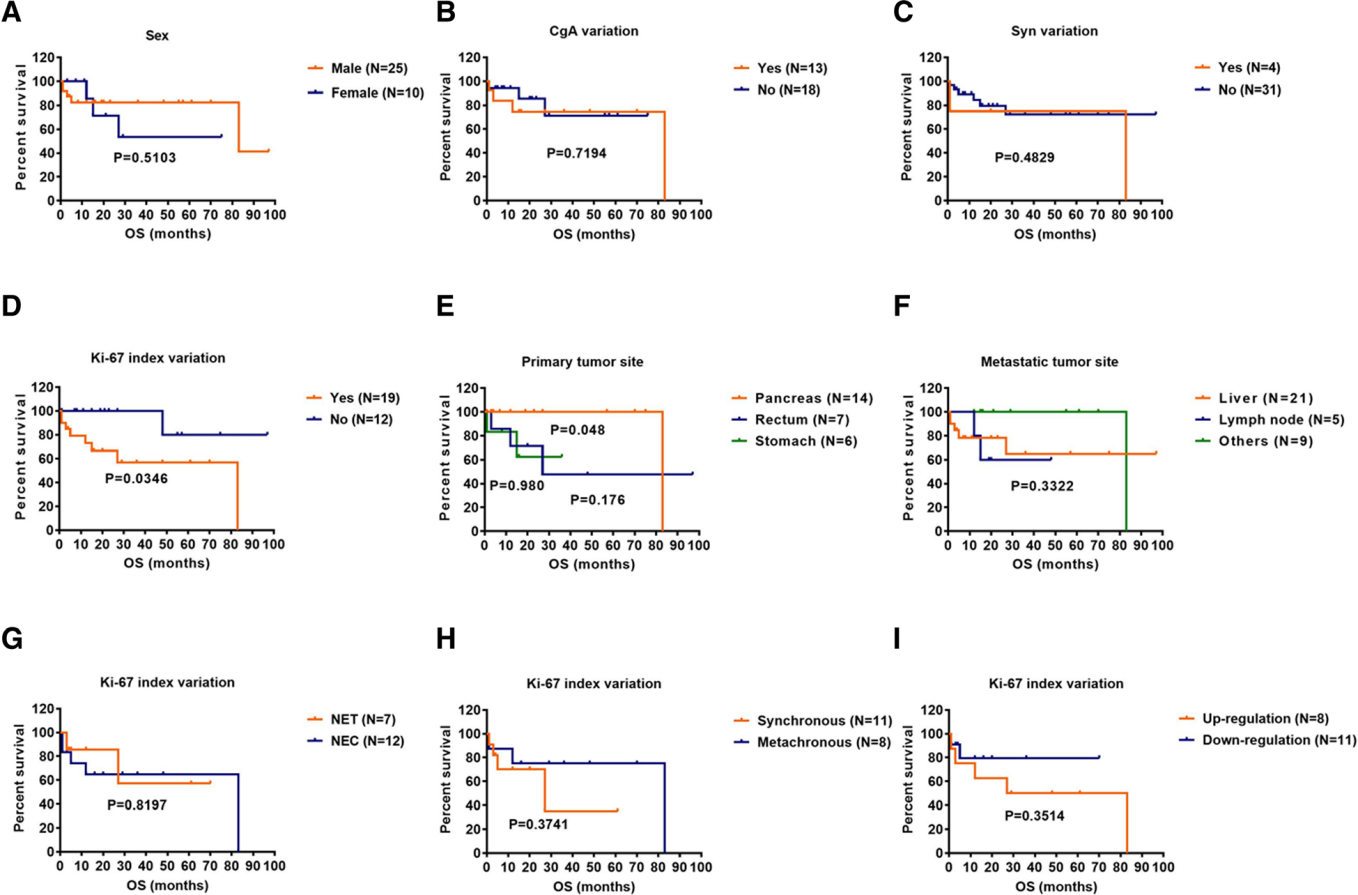
Kaplan-Meier curves for overall survival in patients with multiple pathology specimens according to (**a**) Sex, (**b**) CgA variation, (**c**) Syn variation, (**d**) Ki-67 index variation, (**e**) Primary tumor site, (**f**) Metastatic tumor site. In the case of Ki-67 variation, K-M curves for overall survival in patients with multiple pathology specimens according to (**g**) Classification of NENs, (**h**) Different types of metastases, (**i**) Specific changes of Ki-67 index

**Fig. 6:**
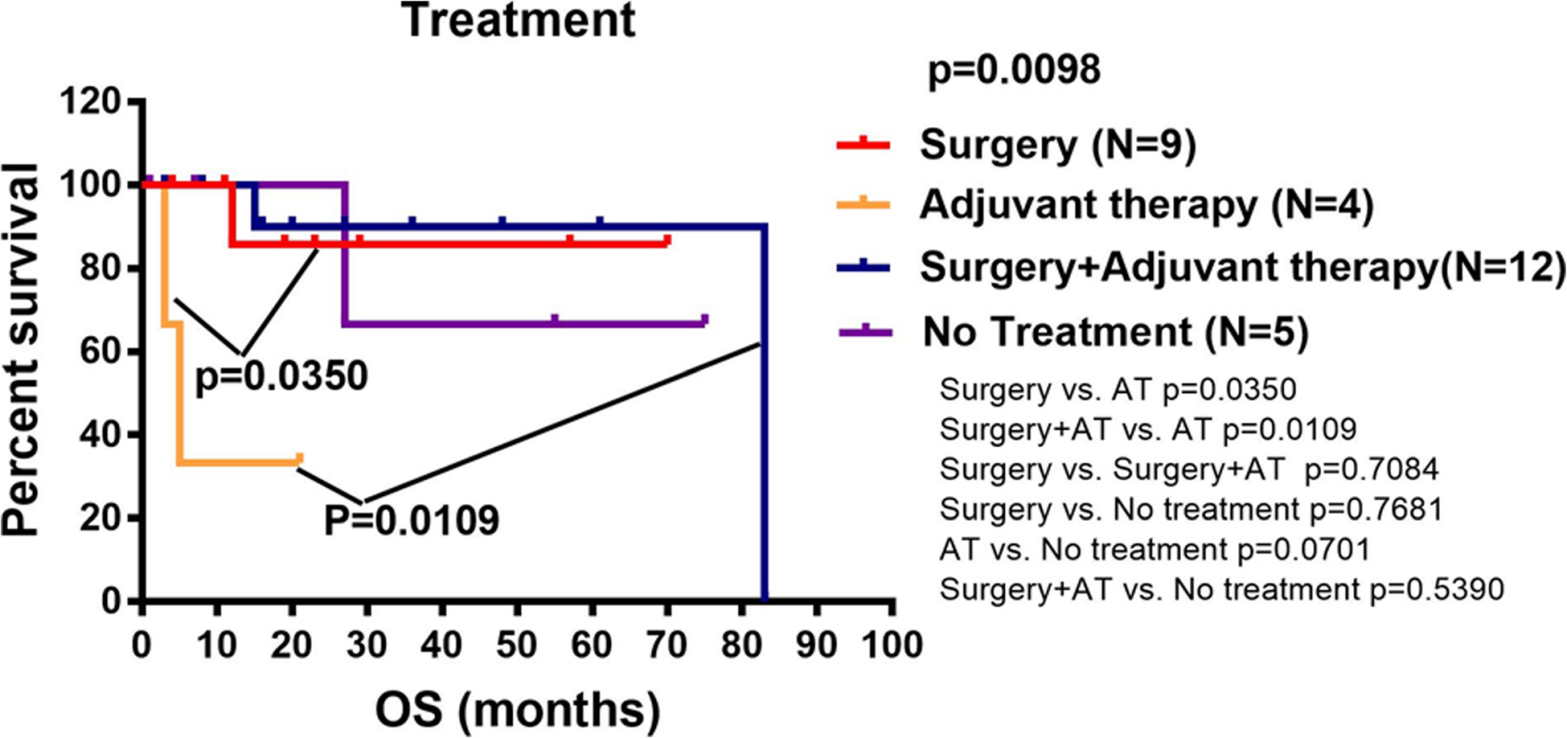
Kaplan-Meier curves for overall survival based on different treatment methods

The Kaplan-Meier survival analysis included sex, CgA variation, Syn variation, Ki-67 index variation, primary tumor site and metastatic tumor site and treatment methods. The results showed that the Ki-67 index variation group had a poorer prognosis than patients in the Ki-67 index non-variation group [hazard ratio 6.800, 95% confidence interval 1.833–25.230; *p* = 0.0346]. For Ki-67 variation group, there was no significant difference in survival time between NET and NEC groups, between synchronous and metachronous metastatic tumor groups, between Ki-67 index up-regulated and down-regulated groups (Fig. 5g-i). To explore the different treatment methods on patient survival, patients were grouped into four groups including surgery, adjuvant therapy, surgery and adjuvant therapy, and no treatment. The results showed that the prognosis of patients receiving surgery or both surgery and adjuvant therapy was better than that of patients who received adjuvant therapy only (*p* = 0.0350, *p* = 0.0109) (Fig. 6).

## Discussion

GEP-NENs represent a heterogeneous family of neoplasms with variable biological and clinical characteristics. Few studies have attempted to assess the heterogeneity of the expression of biological markers in primary and metastatic GEP-NENs. Therefore, in this study, we investigated the pathological heterogeneity of primary and metastatic sites of GEP-NENs and the effect of such heterogeneity of pathological indicators on the prognosis of patients.

In the SEER database, the rectum and small intestine are the most common sites of GEP-NENs [2, 3]. Our results showed that the pancreas, rectum and stomach were the most common sites, which were consistent with the results of a large retrospective single-center study in China [17]. We found that the metastasis rate of patients with GEP-NENs was 39.9%, and the most common site of distant metastasis was the liver. It has been reported that distant metastasis of NENs is a major factor affecting the prognosis [18].

NENs synthesize, store and secrete various peptides and amines [19], which exist in the blood or tissues of patients. These have become important markers for the diagnosis and follow-up of patients with NENs [20]. Among them, CgA and Syn are considered to be the most important biomarkers for the diagnosis of NENs [21, 22]. Our results showed that the positive rates of CgA and Syn in the primary site were 57.5 and 85.4%, respectively. Many studies have shown that the CgA level and Ki-67 index are closely related to NENs metastasis [23–26], and are useful for determining the prognosis [27–30].

Although most studies have explored the relationship between these biological indicators and tumor metastasis and prognosis, little attention has been paid to the heterogeneity of marker expression between primary and metastatic tumor sites, and the impact of such heterogeneity on disease progression has not been fully evaluated. Lindholm et al. [31] found that in small-intestinal NENs, 38.5% showed the grade variation in CgA and 54% showed the grade variation in Syn between the primary and metastatic sites. In this study, 37.1% of cases showed the variation in CgA and 11.4% showed the variation in Syn between the primary and metastatic tumor sites. The variation of CgA was in line with Lindholm’s study, and we found that the variation of CgA in different primary tumor sites was similar, ranging from 42.9 to 50%. We consider the difference may be due to the heterogeneity of NENs at different sites in Syn variation. In our study, the variation rates of Syn were different in different sites of NENs, with the highest variation rate of Syn in rectal NENs (28.6%) while the lowest variation rate of Syn (7.1%) in pancreatic NENs. Due to the high incidence of small-intestinal NENs in Europe and America, Lindholm et al. only focused on small-intestinal NENs, while the incidence of small-intestinal NENs in China was extremely low, so our study focused on NENs in the whole digestive tract. We consider this heterogeneity of Syn variation might be site-related, and further large sample studies are needed to confirm these results.

Singh et al. [32] reported that, in 37.0% of NENs patients, the Ki-67 index changed during the course of the disease, and the WHO classification was upregulated in 27.8% of these patients. Grillo et al. found 39% discrepancy in grade between primary and metastatic tumors [33]. Keck KJ et al. found grade variation occurred in 34% of patients between primary and metastatic tumors [34]. In our study, we found that, in 54.3% of GEP-NENs patients, the Ki-67 index differed between primary and metastatic lesions; as a result, the WHO classification changed in 8.6% of the patients.

In addition, we further analyzed the heterogeneity of CgA, Syn, and the Ki-67 index according to the primary tumor site, and found that these markers differed between the primary and metastatic sites. While there was no significant variation in CgA or Syn in the pancreas, rectum or stomach, the Ki-67 index showed obvious variation, especially in the rectum, where variation was seen in 85.7% of the patients. Interestingly, we also found that CgA and Ki-67 variability were more common in metachronous metastatic tumors than in synchronous metastatic tumors. The Ki-67 variation, in particular, was as high as 80% in metachronous metastases, which was in line with the study, that showed 83% of patients had an increase in Ki-67 rate in the metachronous metastatic site and a change in grade [33]. A Kaplan-Meier survival analysis showed that the overall survival time of patients with the Ki-67 index variation was significantly shortened, which was consistent with the previous report [35], while CgA and Syn variation had no significant correlation with patient survival. The mechanism of this variation in CgA, Syn and the Ki-67 index between primary and metastatic NENs is still unclear. A previous study deduced that inconsistencies in the expression of biomarkers in primary and metastatic tumors may be caused by the heterogeneity of the tumor itself [33, 36], which may affect the clinical treatment [37].

In summary, this study showed that the Ki-67 index showed heterogeneity between the primary and metastatic foci, and that patients with such Ki-67 index variation had a poor prognosis. In contrast, there was little variation in CgA or Syn expression during disease progression, and CgA and Syn variation had no effect on the prognosis. The mechanism that underlies this heterogeneity has yet to be determined. Further studies with a larger sample size will be needed to specifically explore the mechanism of this tumor-marker variation.

## Conclusions

Our results show that there is pathological heterogeneity between the primary and metastatic sites of GEP-NENs. The Ki-67 index shows obvious heterogeneity and patients with such Ki-67 index variation have a poor prognosis. In contrast, there is little heterogeneity in CgA and Syn expression throughout disease progression, and their variations have no effect on the prognosis. Close attention should be given to the clinicopathological heterogeneity between the primary and metastatic lesions to better monitor the progress of NENs and potentially contribute to a more effective treatment.

## Supplementary information

**Additional file 1: Supplementary Table 1.** CgA, Syn and grading in patients between primary and metastatic sites.

## Data Availability

The datasets used and/or analyzed during the current study are available from the corresponding author on reasonable request.

## Abbreviations

CgA: chromogranin A
GEP-NENs: gastroenteropancreatic neuroendocrine neoplasms
MiNEN: mixed neuroendocrine–non-neuroendocrine neoplasm
NECs: poorly differentiated neuroendocrine carcinomas
NENs: neuroendocrine neoplasms
NETs: well-differentiated neuroendocrine tumors
OS: Overall survival
SEER: Surveillance, Epidemiology, and End Results Program
Syn: synaptophysin
WHO: World Health Organization
