# Supplementary material for: Clinicopathological heterogeneity between primary and metastatic sites of gastroenteropancreatic neuroendocrine neoplasm
Source: Diagn Pathol. 2020 Sep 11;15:108. doi: 10.1186/s13000-020-01030-x (PMC7488304; doi:10.1186/s13000-020-01030-x)
Supplement: Supplementary file 1 — Additional file 1: Supplementary Table 1. CgA, Syn and grading in patients between primary and metastatic sites. [file 13000_2020_1030_MOESM1_ESM.doc]

**Supplementary Table 1. CgA，**Syn and grading in patients between primary and metastatic sites

| **Variable** | **Primary tumor**  **Site (n=35, %)** | **Metastatic tumor site (n=35, %)** | ***P* value** |
| --- | --- | --- | --- |
| **CgA** |  |  | 0.633 |
| Positive | 22 (62.9) | 23 (65.7) |  |
| Negative | 10 (28.6) | 8 (22.9) |  |
| **Syn** |  |  | 1.000 |
| Positive | 33 (94.3) | 34 (97.1) |  |
| Negative | 2 (5.7) | 1 (2.9) |  |
| **Grading or classification** |  |  | 0.904 |
| NET G1 | 6 (17.1) | 4 (11.4) | 0.495 |
| NET G2 | 7 (20) | 6 (17.1) | 0.759 |
| NET G3 | 1 (2.9) | 2 (5.7) | 1.000 |
| NEC | 20 (57.1) | 20 (57.1) | 0.760 |

Abbreviations: CgA, chromogranin; Syn, synaptophysin; NET, neuroendocrine tumors; NEC, neuroendocrine carcinoma
